# Supplementary material for: Analysis of HLA Alleles in Different Cohorts of Patients Infected by L. infantum from Southern Spain
Source: Int J Mol Sci. 2024 Jul 27;25(15):8205. doi: 10.3390/ijms25158205 (PMC11311343; doi:10.3390/ijms25158205)
Supplement: Supplementary file 1 [file ijms-25-08205-s001.zip › Supplementary Table S3.pdf]

Supplementary Table S3. Frequency of the different haplotypes in each group.

Haplotype	Leishmania Disease	Asymptomatic infection	Cohabitans	Controls	Infected
**7.1 (HLA-A*03:01, B*07:02, C*07:02, DRB1*15:01, DQB1*06:02)**	0.019	0.009	0.047	0.015	0.012
**7.2 (HLA-A*24:02, B*07:02, C*07:02, DRB1*01:01, DQB1*05:01)**	0	0	0	0.004	0
**8.1 (HLA-A*01:01, B*08:01, C*07:01, DRB1*03:01, DQB1*02:01)**	0.038	0.027	0	0.018	0.031
**18.1 (HLA-A*25:01, B*18:01, C*12:03, DRB1*15:01, DQB1*06:02)**	0.019	0.009	0	0.006	0.012
**18.2 (HLA-A*26:01, B*18:01, C*05:01, DRB1*03:01, DQB1*02:01)**	0	0	0	0.001	0
**18.3 (HLA-A*02:01, B*18:01, C*05:01, DRB1*11:02, DQB1*03:01)**	0.038	0	0.031	0.004	0.012
**35.2 (HLA-A*11:01, B*35:01, C*04:01, DRB1*01:01, DQB1*05:01)**	0	0	0	0.009	0
**35.3 (HLA-A*11/*30, B*35, C*04:01, DRB1*01:01, DQB1*05:01)**	0	0	0	0.002	0
**35.4 (HLA-A*24:02, B*35:08, C*04:01, DRB1*11:03, DQB1*03:01)**	0.019	0.027	0.031	0.009	0.025
**35.5 (HLA-A*01:01, B*35:02, C*04:01, DRB1*11:04, DQB1*03:01)**	0	0	0	0.006	0
**37.1 (HLA-A*02, B*37:01, C*06:02, DRB1*10:01, DQB1*05:01)**	0	0	0	0.002	0
**38.1 (HLA-A*26:01, B*38:01, C*12:03, DRB1*04:02, DQB1*03:02)**	0	0	0	0.002	0
**44.2 (HLA-A*29:02, B*44:03, C*16:01, DRB1*07:01, DQB1*02:02)**	0	0.036	0.016	0.028	0.025
**47.1 (HLA-A*03:01, B*47:01, C*06:02, DRB1*07:01, DQB1*02:02)**	0	0	0	0.001	0
**50.1 (HLA-A*02/*19, B*50:01, C*06:02, DRB1*07:01, DQB1*02:02)**	0	0.009	0	0.006	0.006
**52.1 (HLA-A*24:02, B*52:01, C*12:02, DRB1*15:02, DQB1*06:01)**	0.019	0	0	0.003	0.006
**60.1 (HLA-A*31:01, B*40:01, C*03:04, DRB1*04:04, DQB1*03:02)**	0	0	0	0.002	0
**60.2 (HLA-A*02:01, B*40:01, C*03:04, DRB1*08:01, DQB1*04:02)**	0	0	0	0.002	0
**60.3 (HLA-A*02:01, B*40:01, C*03:04, DRB1*13:02, DQB1*06:04)**	0.019	0	0.031	0.003	0.006
**62.1 (HLA-A*02:01, B*15:01, C*03:04, DRB1*04:01, DQB1*03:02)**	0	0.018	0.031	0.006	0.012
**62.3 (HLA-A*01:01, B*15:01, C*03:03, DRB1*13:01, DQB1*06:03)**	0	0	0.016	0.002	0
**64.1 (HLA-A*02/03, B*14:01, C*08:02, DRB1*07:01, DQB1*02:02)**	0	0	0	0.007	0
**65.1 (HLA-A*33:01, B*14:02, C*08:02, DRB1*01:02, DQB1*05:01)**	0	0.009	0.016	0.01	0.006
**65.2 (HLA-A*03:01, B*14:02, C*08:02, DRB1*13:03, DQB1*06:09)**	0	0.009	0	0.002	0.006
